# The role of nitric oxide and endothelin on optic nerve head blood flow autoregulation

**DOI:** 10.1186/2050-6511-13-S1-A28

**Published:** 2012-09-17

**Authors:** Doreen Schmidl, Agnes Boltz, Semira Kaya, René Werkmeister, Reinhard Told, Stefan Palkovits, Gabriele Fuchsjäger-Mayrl, Gerhard Garhöfer, Leopold Schmetterer

**Affiliations:** 1 Department of Clinical Pharmacology, Medical University of Vienna, 1090 Vienna, Austria; 2 Center for Medical Physics and Biomedical Engineering, Medical University of Vienna, 1090 Vienna, Austria; 3 Department of Ophthalmology and Optometry, Medical University of Vienna, 1090 Vienna, Austria

## Background

Autoregulation is defined as the ability of a vascular bed to keep its blood flow constant despite changes in perfusion pressure. While several studies have investigated choroidal blood flow regulation, only few data are available for the optic nerve head (ONH). The aim of the present study was to explore the potential role of a potent vasodilator (nitric oxide) and a potent vasoconstrictor (endothelin-1) in ONH autoregulation.

## Methods

Two randomized, double-blind, placebo-controlled, cross-over studies were performed. Eighteen subjects received either a nitric oxide synthase (NOS) inhibitor (L-NMMA) or placebo. Fifteen subjects received either an endothelin ET_A_ receptor antagonist (BQ-123) or placebo on two trial days. Isometric exercise (squatting) was performed to increase ocular perfusion pressure (OPP). ONH blood flow (ONHBF) was measured continuously by means of laser Doppler flowmetry. OPP was calculated as ⅔ × (mean arterial pressure) − (intraocular pressure).

## Results

During all experiments the response in ONHBF was less pronounced than the response in OPP indicating autoregulation. L-NMMA had no influence on the response of ONHBF to isometric exercise (p = 0.27). When BQ-123 was administered the increase in ONHBF during squatting was more pronounced than during placebo (p < 0.01) leading to a left-shift of the pressure/flow curve.

## Conclusions

The present data confirm previously published observations that ONHBF shows some autoregulatory capacity during changes in OPP. Nitric oxide does not seem to be involved in the regulatory mechanisms during isometric exercise. In contrast, endothelin-1 seems to provide some of the vasoconstrictor tone that counteracts the increase in OPP during isometric exercise.

